# Inequities in the National Clinical Assessment Tool for Medical Students in the Emergency Department

**DOI:** 10.5811/westjem.43506

**Published:** 2025-10-03

**Authors:** Bushra Z. Amin, C. Jessica Dine, Erica R. Tabakin, Michael Trotter, Janae K. Heath

**Affiliations:** * Perelman School of Medicine at the University of Pennsylvania, Philadelphia, Pennsylvania; † Hospital of the University of Pennsylvania, Department of Medicine, Philadelphia, Pennsylvania; ‡ Hospital of the University of Pennsylvania, Department of Emergency Medicine, Philadelphia, Pennsylvania; § Leonard Davis Institute of Economics at the University of Pennsylvania, Philadelphia, Pennsylvania

## Abstract

**Introduction:**

The National Clinical Assessment Tool for Emergency Medicine (NCAT-EM) was designed to standardize medical student assessments during emergency medicine clinical rotations. While multiple assessment tools implemented in medical education have been prone to inequities, it remains unknown how student and rater demographics impact NCAT-EM scores. In this study we examined how a student’s gender and status as under-represented in medicine (URM) affected NCAT-EM scores.

**Methods:**

This was a retrospective cohort study of all NCAT-EM assessments of clerkship medical students at a single institution in 2022. We performed mixed-effect ordinal logistic regression analyses to determine the association between the seven NCAT-EM domains (history/physical, prioritized differential, formulation of plans, observation/monitoring, emergency management, communication, and global assessment) and student gender, as well as the NCAT-EM domains and students’ URM status (specifically in domains of race and ethnicity). We adjusted our analyses for the site of rotation, time, the rater’s role (attending or resident), and rater demographics (gender, URM status). We then evaluated the interaction in gender concordance and URM-status concordance on outcomes.

**Results:**

A total of 1,881 NCAT-EM assessment forms were submitted on 142 students completed by 266 raters. There were no significant associations between student gender and NCAT-EM ratings across the seven domains. We found an association between URM students and lower scores in multiple NCAT-EM domains, including global assessment (odds ratio [OR] 0.50, CI 0.25–0.99, *P* = .01); history/physical (OR 0.38, CI 0.19–0.77, *P* = .01); and prioritized differential (OR 0.47, CI 0.26–0.88, *P* = .02). This effect was moderated by a significant positive interaction effect with URM concordance between raters and students in the prioritized differential and observation/monitoring domains.

**Conclusion:**

This is the first study to highlight differences in both gender and status as under-represented in medicine within the nationally implemented NCAT-EM assessment tool. Women students were overall rated similarly across the NCAT-EM domains compared to men, with no association of gender on ratings. However, students’ URM status was associated with lower scores in multiple NCAT-EM domains. This finding was mitigated by URM concordance between faculty and resident raters. Our findings support the need for additional studies to understand bias and inequities in the application of the NCAT-EM tool nationally.

## INTRODUCTION

A longstanding challenge in medical education has been to accurately assess medical students on clinical rotations with assessment tools that have strong validity and reliability evidence.^1^,^2^ The fairness and accuracy of clinical assessment of medical students is critical as it informs clinical grades, Medical Student Performance Evaluation (MSPE), or “dean’s letters”, and—for those applying to emergency medicine (EM) residency—the Standardized Letter of Evaluation (SLOE).^3^ As EM residency program directors have consistently ranked SLOEs and EM rotation grades as some of the most important criteria when offering interviews and ranking applicants,^4^ ensuring fairness and accuracy in these assessments plays a paramount role in achieving equity among EM applicants.

In an effort to improve the fairness and accuracy of EM medical student assessments (and thus that of SLOEs and EM rotation grades), the National Clinical Assessment Tool for Emergency Medicine (NCAT-EM) was developed in 2016 via consensus as a standardized assessment tool.^5^ This tool allowed for post-shift assessment of students by faculty or residents across six clinical performance domains: history and physical exam skills; prioritized differential diagnosis; ability to formulate a plan; observation, monitoring, and follow-up; emergency recognition and management; and patient- and team-centered communication. This tool has begun to replace institution-specific tools in numerous EM rotations across the United States,^2^,^5^,^6^ and was the first nationally standardized, specialty-specific, entrustable professional activities-based assessment tool for medical students.^5^

Prior work evaluating early implementation of the NCAT-EM suggests it is achieving some of its stated goals. Specifically, Hiller et al noted high internal consistency in scores within a given institution,^6^ suggesting that this tool supports reliable comparison of students within an institution during the residency application process. However, this work showcased some gaps in the validity evidence, specifically noting site-specific variation in ratings, perhaps suggesting limitations in the response process of the validity (how the raters differentially use the tool) or the generalizability of the tool.^1^ Additionally, this prior work was predominantly limited to medical students in their fourth year (with a high percentage interested in EM residency), limiting generalizability. Additionally, although they found site-specific differences based on student and rater gender, they were unable to examine the association of race or ethnicity on NCAT-EM scores (despite known racial disparities in other standardized EM assessments,^7^,^8^ such as the SLOE^9^–^11^). Additional work to investigate the various domains of validity of this tool would add to the literature.

Unfortunately, such disparities in assessment have been observed throughout medical education,^12^ potentially contributing to the known leadership disparities and pay-based disparities based on gender and race.^13^–^18^ Studies have identified gender- and race-based differences in language used in the MSPE^19^,^20^ and language used in clerkship evaluations.^7^,^19^,^21^ Gender- and race-based differences have also been observed through clinical grades (with lower clinical grades for non-White students, even after adjusting for variables such as scores on Step 1 of the US Medical Licensing Exam)^7^,^22^ and overall recommendations on SLOEs.^9^–^11^ These differences likely represent inequities, especially when the observed variations are not explained by the student performance but instead by other factors such as the clinical learning environment or the evaluator.^23^,^24^ It is not known whether such differences persist despite the implementation of a nationally standardized tool such as the NCAT-EM.

**Population Health Research Capsule**

What do we already know about this issue?

*Clinical assessment tools in medical education often show racial and sex disparities in scoring and narrative feedback*.

What was the research question?

*Do student and rater demographics affect National Clinical Assessment Tool for Emergency Medicine (NCAT-EM) scores in emergency medicine clerkships?*

What was the major finding of the study?

*Under-represented in medicine (URM) students had lower global scores (OR 0.50, 95% CI 0.25–0.99, P =.048), which was mitigated by URM rater concordance*.

How does this improve population health?

*Identifying disparities in clerkship assessments supports equitable evaluation, critical for building a diverse physician workforce*.

To address this gap, our goal in this study was to analyze the association between student and rater demographics and NCAT-EM scores of clerkship students rotating through various emergency department sites at a single institution. Given prior evidence suggesting that concordance in demographics may impact ultimate evaluation,^25^,^26^ we similarly assessed how concordance in student and rater demographics was associated with NCAT-EM scores.

## METHODS

### Setting and Participants

We performed a retrospective, single-center cohort study of all electronically completed NCAT-EM assessments of clerkship medical students at the University of Pennsylvania. Individualized NCAT-EM forms were made available electronically through a Qualtrics (Qualtrics International Inc, Provo, UT) QR code. The dataset included all submitted NCAT-EM assessments from January–December 2022, as assessments completed during this year were unaffected by the COVID-19 pandemic. Given our interest in the impact of demographics on NCAT-EM scores, we excluded assessments for which demographic information (for either the student or the rater) was unavailable.

The Perelman School of Medicine curriculum includes 1.5 years of pre-clerkship content, followed by a one-year clerkship year, consisting of eight core clerkships (emergency medicine, family medicine, internal medicine, neurology, obstetrics and gynecology, pediatrics, psychiatry, and surgery) graded on an honors/high pass/pass/fail basis. During the clerkship year, students additionally complete an additional month of otolaryngology, orthopedic surgery, anesthesia, and ophthalmology (one week each, graded on a pass/fail basis). The EM clerkship is a four-week core clerkship completed at either one or two of six affiliated clinical sites.

The NCAT-EM has been used for clinical assessment of EM clerkship students in this institution since 2018. The NCAT-EM consists of six clinical performance domains rated on a four-point entrustability scale, a global assessment domain, a professionalism section, and mandatory free-text comments for strengths and suggestions for improvement (Supplemental Table 1). Students are required to present a QR code linking the NCAT-EM to attendings or EM residents during every shift, which is completed at the rater’s convenience on an online platform.

During the EM clerkship, students were assigned to work 14 eight-hour shifts (or total hourly equivalent for sites with 10- or 12-hour shifts) for the duration of the clerkship. Students were required to present the QR code to at least one rater per shift (either an attending or a supervising resident ranging from postgraduate year [PGY] 2–4). The dataset did not include discrete PGY-level data for resident raters. Prior to and during this study, the clerkship directors performed annual education focused on the NCAT-EM tool and the process of assessment, which consisted of an introduction to the tool, a brief overview of the scale, and a review of medical student evaluation processes. This information session included both faculty and residents at all sites (for all individuals who would be working with students).

### Data Collection and Analysis

Data collected in addition to completed NCAT-EM forms included student factors (gender and under-represented in medicine [URM] status) and rater factors (gender, URM status, and role, either resident or faculty). We extracted gender and race data for students from admissions demographics based on self-identification. For faculty, self-reported gender and race were obtained through the university’s faculty affairs database. We defined URM status for both faculty and students using the Association of American Medical Colleges (AAMC) definitions. The URM status was specifically chosen as a binary variable (as opposed to race and ethnicity data), to improve power in our statistical analysis. Importantly, the definition of URM can broadly include groups that are minoritized, such as first-generation, low-income students, or students with disabilities, although for this work we used the AAMC definition of URM based on race and ethnicity. The dataset also included the quarter of year in which the student was completing the clerkship (block 1, 2, 3, or 4), and the clinical site where they were rotating. All data were deidentified prior to data analysis.

We performed univariate ordinal logistic regression analyses to determine the association between the global assessment on the NCAT-EM tool (bottom third, middle third, top third, or top 10%), with student gender, student URM status, faculty gender, faculty URM status, clerkship site, and rotation block. We then performed mixed-effect ordinal logistic regression analyses to determine the association between NCAT-EM scores and student gender, clustered by student, after adjusting for site of rotation, time, role of rater, student URM status, and rater demographics (gender, URM status). To assess the association with URM status, we performed mixed-effect ordinal logistic regression analyses to determine the association between NCAT-EM scores and student URM status, clustered by student, after adjusting for site of rotation, block, role of rater, student gender, and rater demographics. Given the hypothesis that concordance in rater gender and student gender and URM status might be associated with NCAT-EM scores, we also assessed the interaction between student gender and rater gender, and student URM status and rater URM status.

For each analysis, the model was clustered on student (random effects) and rater (random effects). This model was used intentionally to adjust for the non-independent nature of students and/or raters throughout the dataset, as this model provides adjusted standard errors accounting for student and/or rater clustering (random effects) throughout the dataset. Based on prior factor analyses showing each domain in the NCAT-EM assessed unique domains, we repeated the above analysis for each of the six clinical performance domains of the assessment. (See Supplemental Table 1 for the NCAT-EM domains.)

While our primary analysis included URM (as per AAMC definition) as a binary variable, we aimed to further understand our findings in the context of URM categories, recognizing that URM individuals who spanned different identities might have had different experiences with assessment. Thus, we performed a sensitivity analysis using racial and ethnic groups within the AAMC definition of URM (African-American/Black, Hispanic/Latino, Native American, including American Indian, Alaska Native, and Native Hawaiian, Pacific Islander, and mainland Puerto Rican). We then performed a second sensitivity analysis comparing individuals identifying as Black compared to other individuals (noting the large proportion of individuals within the cohort identifying as URM were Black and the distinct experiences of this population^27^,^28^).

We completed statistical analysis using STATA v18.0 (StataCorp, LLC, College Station, TX). Statistical significance was determined using a *P*-value of .05 (not adjusting for multiple comparisons given the exploratory nature of the analysis, to reduce the risk of type 2 error). This study was deemed exempt by the University of Pennsylvania Institutional Review Board.

## RESULTS

Over the course of 2022, 1,881 complete NCAT-EM assessment forms were submitted on 142 distinct students (consisting of 74 women [52%] and 68 men [48%], including 34 [24%) who identified as URM) completed by 266 different raters. We excluded 122 NCAT-EM forms prior to analysis, due to incomplete demographic information for the rater. The median number of completed forms per student was 13 [(IQR 11–15), which was similar between genders (13.4 for men vs 13.1 for women, *P* = .59). There were fewer NCAT-EM assessments completed on those who identified as URM within the sample (with a mean of 11.8 vs 13.8 assessments for non-URM students, *P* = .01). Most assessments were completed by raters who identified as men (*n* = 1,070, 60%), and 11% (*n* = 195) were completed by raters who identified as URM. The racial demographics of faculty identified as URM (as per AAMC definitions) was 119 (61%) Black, 55 (28%) Hispanic or Latino, and 21 (11%) Pacific Islander. The racial demographics of students identified as URM (as per AAMC definitions) was 224 (56%) Black, 38 (10%) Hispanic or Latino, and 138 (35%) spanning multiple groups. Complete demographic information of completed NCAT-EM forms are included in Table 1.

Distribution of scores for each of the six clinical performance domains on the NCAT-EM as well as the global assessment domain (see Supplemental Table 1) are summarized in Table 2. Global assessment scores were skewed leftward (consistent with prior national data^5^), with 38 ratings (2.1) ratings representing the lower third in the global assessment, 506 (28%) in the middle third, 878 (49%) in the upper third, and 387 (21%) in the top 10% (“exceptional”).

The results of the univariate ordinal logistic regression are shown in Table 3. In the univariate analysis, there was a significant association based on rater role, with faculty raters being less likely to rate students in the higher entrustment scores compared to resident raters for all domains (*P* < .001 for all domains); thus, this was included in the multivariate analysis. There was also an association between rotation block and NCAT-EM scores, and site of the rotation and NCAT-EM scores; thus, these variables were included in the final regression model.

The results of the multivariate ordinal logistic regression, clustered by student and rater, are shown in Table 4, using a significant threshold of *P =* .05 (rather than adjusting for multiple comparison due to the exploratory nature of the study). As there was no significant interaction between the student gender and rater gender in each analysis, this interaction term was excluded from the final regression model. The final regression model included the rater gender and student gender, rater and student URM status (and the interaction between them), rater role (faculty vs resident), clinical site, and rotation block. The results of the mixed regression identified no significant associations between student gender and NCAT-EM scores for each NCAT-EM domain (Table 4). There was a significant interaction effect between student gender and rater gender in the domain of history and physical exam (OR 0.31, CI 0.11–0.83, *P* = .02). There were no other significant interaction effects in the remainder of the domains.

Student URM status was associated with lower scores for the global assessment, (OR 0.50, CI 0.25–0.99, *P* = .05), history/physical exam domain (OR 0.38, CI 0.19–0.77, *P* = .01) and the prioritized differential diagnosis domain (OR 0.47, CI 0.26–0.88, *P* = .02) after multivariate adjustment, as shown in Table 4. These findings were moderated by a significant positive interaction effect between student and rater URM status in the observation/monitoring domain (OR 4.55, CI 1.21–17.1, *P* =.03), suggesting that concordance in URM status between raters and students lessened (and in some instances, reversed) the negative effect of URM status on NCAT-EM scores. More specifically, when assessing the significant domains for a given student, the adjusted ORs for URM-concordant dyads were adjusted OR 0.83 for the global assessment (suggesting the difference persisted); adjusted OR 1.06 for history/physical domains; and adjusted OR 1.76 for observation/monitoring (suggesting reversal of the score, and URM-concordant dyads had higher odds of receiving a higher score than the reference cohort).

The sensitivity analysis using racial and ethnic groups within the URM status (Black, Hispanic/Latino, Native Americans, including American Indians, Alaska Natives, and Native Hawaiians, Pacific Islanders, and mainland Puerto Ricans demonstrated a poorer fit to the data—based on likelihood ratio tests and Bayesian information criterion and Akaike’s information criterion comparisons—than the original model. As this approach risked underestimating the true effect, it was not included in the results. In our sensitivity analysis comparing individuals who were Black to other individuals in the cohort, we found significant associations with NCAT-EM ratings in the domains of history/physical domain (OR 0.32, CI 0.14–0.76, *P* = .01); prioritized differential domain (OR 0.36, CI 0.17–0.79, *P* =.01); the ability to formulate a plan domain (OR 0.46, CI 0.59–1.48, *P* =.05), with significant interaction effects noted in the majority of domains. (See Table 5 for full details.)

## DISCUSSION

Our study demonstrates important associations between both rater and student demographics and NCAT-EM scores within our cohort, with notable findings based on student URM status. Our multivariate analysis did not find any gender-related differences in NCAT-EM domains. However, the multivariate analysis showed that students identified as URM received lower NCAT-EM scores in several domains, including the history/physical exam domain and the prioritized differential diagnosis domain. This effect was mediated (and in some cases reversed) by concordance of URM status between raters and students in some of the domains, such that concordance in URM status between students and raters was associated with higher NCAT-EM scores.

The association between student URM status with lower NCAT-EM scores is consistent with prior literature documenting longstanding racial disparities in clerkship grading.^21^,^29^,^30^ Despite the NCAT-EM being noted to have excellent internal consistency based on prior studies,^6^ this suggests that the use of the tool continues to be impacted by its differential use by raters and raises some concern about additional domains of validity with the tool.^1^ It is important for us as medical educators to ensure the assessment tool widely used to guide clerkship grading does not introduce any construct irrelevance variance at all.^31^,^32^

In this study, the observed score differences by URM status may be the result of implicit bias of raters affecting both their global perception and perception of competency-related behaviors of students, lack of mentorship leading to inequitable opportunities, or different lived experiences of URM students impacting their experience, and performance in the clinical environment (including stereotype threat, microaggressions, patient mistreatment, being tasked with being a racial ambassador, unrewarded labor, limited resources, and othering).^17^,^21^,^33^–^35^ The complexity of this amalgam of factors that exacerbates disparities in clerkship grading has been described as the “social milieu of medical education,”^35^ and may ultimately contribute to the inequities observed in other standardized assessments used in EM, such as the SLOE. Improved understanding of these disparities in the EM clerkship setting and further evaluation of the validity evidence of the NCAT-EM tool is critical to identify solutions to mitigate these issues.

Perhaps more interestingly, some of the findings of a differential score based on URM status were mitigated (and in some cases, reversed) by URM concordance between the student and rater, specifically within the prioritization of a differential, and the observation and monitoring domains. As a possible explanation, URM concordance may reduce implicit bias of the rater as well as the other effects of racism on the medical student, such as stereotype threat. Concordance between the rater and student can also enhance performance of the student through the role-model effect.^33^ This aligns with prior studies that have shown the importance of racial concordance in multiple domains, including patient care, professional development,^17^ and medical education assessment,^36^,^37^ further highlighting the critical nature of supporting equity initiatives to advanced diversity across EM faculty and residents.

It is not clear why this phenomenon would be present for only two of the NCAT-EM domains, although it could represent something unique about those domains, including that they may capture more direct interaction between students and raters (such as prioritizing differentials) and, thus, concordance would be more heavily impacted. Regardless, this further suggests that additional robust validity studies of the NCAT-EM tool are needed. Additionally, while we noted various impacts of URM concordance on the NCAT-EM scores, the impact on the overall disparities identified in our study may be negligible, and further work is needed to examine this phenomenon across a larger sample of more diverse learners and raters.

Another interesting observation within our study was that URM students had fewer submitted NCAT-EM forms in the full cohort, which persisted after adjusting for minor site differences. The structure of the NCAT-EM within our institution requires learners to seek out designated feedback and collect assessments via a QR code. This difference could indicate differences in self-promotion behavior,^38^ which may uniquely disadvantage URM students. Specifically, there is a complex interplay between evaluations, biases, and the associated impact on confidence, self-esteem, and motivation. In URM students, negative evaluations, even if biased, may reinforce stereotype threat—defined as a fear of confirming negative stereotypes about their group—17 and ultimately hinder professional growth. Understanding this, as well as other unique barriers to seeking evaluation by URM students as observed in this study, should be further evaluated.

We also found no difference in NCAT-EM scores based on gender, with no significant difference in scores between men and women in the cohort. This absence of gender associations across the NCAT-EM performance domains was surprising, and in contrast to prior work analyzing the NCAT-EM in medical students. Specifically, in a study by Hiller and colleagues, there were student gender-based differences in composite NCAT-EM scores at 4 of the 13 sites included in their study.^6^ However, this prior work was conducted with limited demographic data and an over-representation of male students, with predominantly students in their final year of medical school. It is possible that gender disparities across diverse assessment domains become apparent at later stages in training (as has been observed in residency assessments).^39^,^40^

As NCAT-EM scores inform clinical grades, and subsequently the SLOE and MSPE, it is critical to mitigate disparities in use of the NCAT-EM tool as found in our study. The NCAT-EM has features of prior recommendations to reduce grading inequity, including workplace-based assessment, criterion-based rubrics, and competency-based grading. Our data show that rating disparities are still present, despite high internal consistency metrics of the NCAT-EM. This argues that the tool itself does not contribute to disparities, but real-world use of the tool by raters contributes to these differences. Ultimately, the scoring differences found in our study support the use of rater training, which has been shown to improve the accuracy of workplace-based entrustment ratings of medical learners.^41^

Additionally, ongoing efforts to promote an equitable and diverse workforce are necessary, noting the role of concordance on some of these disparities. Ultimately, achieving fairness and accuracy in NCAT-EM assessments is crucial to promoting gender and racial equity among EM applicants, especially with national implementation of the NCAT-EM tool. In addition, clerkships in other specialties should note that despite the positive impacts of using a standardized and national assessment instrument with strong reliability, testing by itself is not the solution for overcoming observed differences not explained by student performance.

## LIMITATIONS

Although we identified compelling findings, this study had several limitations. Our study analytic approach involved multiple regressions, and we did not adjust for multiple comparisons due to the exploratory nature of the study, which increased the risk of type 1 error in our conclusions. However, we feel the presence of findings in our sensitivity analysis suggests that this finding is a true trend. In addition, the study was limited to a single institution. Although it included six different sites within the institution (each with a unique culture and patient population), obtaining a multicenter study across distinct geographical regions is an important next step to fully evaluate the effect of student and rater demographics on NCAT-EM performance nationally. Furthermore, it is not yet clear whether these findings among second- and third-year clerkship students can be generalized to more senior medical students on sub-internships or electives, and additional work evaluating the impact of URM status on advanced students is needed. Our study did not include individuals who identified as gender diverse, which would also be important to include in future research. Finally, while we importantly noted some differences in NCAT-EM use by site and by level (residents NCAT-EM scores were higher as compared to faculty), we were unable to assess PGY level in residency training nor the impact of faculty development on the tool. This is an important area of future work.

## CONCLUSION

While we found no association between student gender with NCAT-EM scores, we did find an association between student under-represented in medicine status in two of six NCAT-EM performance domains, an effect that was mediated by concordance in URM status with the rater. Future multi-institution research is needed to verify grading disparities based on student and rater characteristics on the national level, which would further support the use of multifaceted interventions to mitigate disparities in ratings, including diversity efforts in recruitment practices, equitable access to medical school resources, gender- or URM-specific student support and rater training to ultimately promote equity among emergency physicians.

## Supplementary Information







## Figures and Tables

**Table 1:** Student characteristics, rater characteristics, clinical site and block of completed NCAT-EM assessment forms.

Characteristic	Completed NCAT-EM forms (n, %)
Student gender	1881 (100.0%)
Men	913 (48.5%)
Women	968 (51.5%)
Student URM status	1881 (100.0%)
URM	400 (21.3%)
African American/Black	224 (56%)
Hispanic/Latino	38 (10%)
Multiple AAMC URM Groups	138 (35%)
Non-URM	1481 (78.7%)
Rater Role	1775 (100.0%)
Resident	484 (27.3%)
Faculty	1291 (72.7%)
Rater Gender	1783 (100.0%)
Men	1070 (60.0%)
Women	713 (40.0%)
Rater URM Status	1759 (100.0%)
URM	195 (11.1%)
African American/Black	119 (61%)
Hispanic/Latino	55 (28%)
Pacific Islanders	21 (11%)
Non-URM	1564 (88.9%)
Clinical Site	1880 (100.0%)
Quaternary care site	694 (36.9%)
Community site A	662 (35.2%)
Community site B	187 (9.9%)
Community site C	129 (6.9%)
Pediatric site	121 (6.4%)
Veteran’s Affairs	87 (4.6%)
Block	1881 (100.0%)
1	569 (30.2%)
2	569 (30.2%)
3	305 (16.2%)
4	438 (23.3%)

All results are expressed as number of NCAT assessment forms completed within each category, followed by percent of assessments for which data are available.

*NCAT-EM*, National Clinical Assessment Tool for Emergency Medicine; *URM*, under-represented in medicine; *AAMC*, Association of American Medical Colleges.

**Table 2:** Overview of ratings for National Clinical Assessment Tool for Emergency Medicine clinical performance domains and global assessment.

NCAT-EM Domain	n (%)
Focused history and physical exam skills	1,879 (100%)
Unable to assess	25 (1%)
Pre-entrustable	118 (6%)
Mostly entrustable	742 (40%)
Fully entrustable/Milestone 1	754 (40%)
Outstanding/Milestone 2	240 (13%)
Ability to generate a prioritized differential diagnosis	1,866 (100%)
Unable to assess	31 (2%)
Pre-entrustable	152 (8%)
Mostly entrustable	818 (44%)
Fully entrustable/Milestone 1	664 (36%)
Outstanding/Milestone 2	201 (11%)
Ability to formulate plan (diagnostic, therapeutic, disposition)	1,861 (100%)
Unable to assess	34 (2%)
Pre-entrustable	163 (9%)
Mostly entrustable	894 (48%)
Fully entrustable/Milestone 1	592 (32%)
Outstanding/Milestone 2	178 (10%)
Observation, monitoring, and follow-up	1,861 (100%)
Unable to assess	42 (2%)
Pre-entrustable	120 (6%)
Mostly entrustable	683 (37%)
Fully entrustable/Milestone 1	747 (40%)
Outstanding/Milestone 2	263 (14%)
Emergency recognition and management	1,851 (100%)
Unable to assess	385 (21%)
Pre-entrustable	98 (5%)
Mostly entrustable	673 (36%)
Fully entrustable/Milestone 1	523 (28%)
Outstanding/Milestone 2	172 (9%)
Patient- and team-centered communication	1,848 (100%)
Unable to assess	40 (2%)
Pre-entrustable	65 (4%)
Mostly entrustable	605 (33%)
Fully entrustable/Milestone 1	794 (43%)
Outstanding/Milestone 2	344 (19%)
Global assessment	1,809 (100%)
Lower third	38 (2%)
Middle third	506 (28%)
Top third	878 (49%)
Exceptional (top 10%)	387 (21%)

All results are expressed as number of NCAT-EM assessment forms completed within each category, followed by percentage of assessments for which data are available.

*NCAT-EM*, National Clinical Assessment Tool for Emergency Medicine.

**Table 3:** Univariate associations between ratings for National Clinical Assessment Tool for Emergency Medicine domains and student and rater characteristics.

	Global Assessment		History/Physical		Prioritized Differential		Ability to Formulate Plan		Observation/ Monitoring		Emergency Management		Communication	

Variable	OR (95% CI)	*P*	OR (95% CI)	*P*	OR (95% CI)	*P*	OR (95% CI)	*P*	OR (95% CI)	*P*	OR (95% CI)	*P*	OR (95% CI)	*P*
Student gender														
Men	1.0 (ref)	-	1.0 (ref)	-	1.0 (ref)	-	1.0 (ref)	-	1.0 (ref)	-	1.0 (ref)	-	1.0 (ref)	-
Women	0.91 (0.77, 1.08)	.30	0.85 (0.72, 1.01)	.06	0.91 (0.77, 1.08)	.30	0.90 (0.76, 1.07)	.24	0. 97 (0.83, 1.15)	.75	1.05 (0.88, 1.26)	.59	0.97 (0.82, 1.15)	.73
Student URM-status														
Non-URM	1.0 (ref)	-	1.0 (ref)	-	1.0 (ref)	-	1.0 (ref)	-	1.0 (ref)	-	1.0 (ref)	-	1.0 (ref)	-
URM	0.91 (0.73, 1.12)	.37	0.90 (0.73, 1.11)	.23	0.92 (0.75, 1.14)	.45	0.67 (0.37–1.22)	.19	1.00 (0.81, 1.23)	.99	.99 (0.81, 1.21)	.93	1.11 (0.90, 1.36)	.33
Rater role														
Resident	1.0 (ref)	-	1.0 (ref)	-	1.0 (ref)	-	1.0 (ref)	-	1.0 (ref)	-	1.0 (ref)	-	1.0 (ref)	-
Faculty	0.46 (0.38, 0.57)	< .001	0.34 (0.28, 0.42)	< .001	0.29 (0.24, 0.36)	<.001	0.38 (0.31, 0.46)	< .001	0.32 (0.27, 0.40)	< .001	0.43 (0.35, 0.53)	<0.001	0.19 (0.15, 0.24)	< .001
Rater gender														
Men	1.0 (ref)	-	1.0 (ref)	-	1.0 (ref)	-	1.0 (ref)	-	1.0 (ref)	-	1.0 (ref)	-	1.0 (ref)	-
Women	1.05 (0.88, 1.26)	.60	1.26 (1.06, 1.51)	.01	1.21 (1.01, 1.44)	.04	1.21 (1.02, 1.45)	.03	1.24 (1.04, 1.48)	0.02	0.86 (0.72, 1.02)	0.09	1.04 (0.87, 1.24)	.65
Rater URM-status														
Non-URM	1.0 (ref)	-	1.0 (ref)	-	1.0 (ref)	-	1.0 (ref)	-	1.0 (ref)	-	1.0 (ref)	-	1.0 (ref)	-
URM	0.67 (0.53, 0.93)	.02	1.14 (0.87, 1.50)	.33	1.23 (0.93, 1.61)	.13	1.29 (0.97, 1.69)	.08	1.37 (1.04, 1.79)	.02	0.95 (0.72, 1.26)	.74	1.08 (0.82, 1.43)	.58

*OR*, odds ratio; *NCAT-EM*, National Clinical Assessment Tool for Emergency Medicine; *URM*, under-represented in medicine.

**Table 4:** Multivariate associations between ratings for National Clinical Assessment Tool for Emergency Medicine domains and student sex and under-represented in medicine (URM) status, after adjusting for rater sex and URM status, concordance of student-rater URM status, clinical site, and time.

	Global assessment		History/physical		Prioritized differential		Ability to formulate plan		Observation/ monitoring		Emergency management		Communication	

Variable	OR (95% CI)	P	OR (95% CI)	P	OR (95% CI)	P	OR (95% CI)	P	OR (95% CI)	P	OR (95% CI)	P	OR (95% CI)	P
Student Sex														
Men	1.0 (ref)	-	1.0 (ref)	-	1.0 (ref)	-	1.0 (ref)	-	1.0 (ref)	-	1.0 (ref)	-	1.0 (ref)	-
Women	0.93 (0.54, 1.60)	.78	0.84 (0.50, 1.42)	.52	0.98 (0.61, 1.57)	.93	0.95 (0.60, 1.51)	.84	1.26 (0.84, 1.89)	.26	0.87 (0.60, 1.27)	.48	1.21 (0.82, 1.78)	.35
Interaction between rater and student gender														
Sex concordance between men	1.0 (ref)	-	1.0 (ref)	-	1.0 (ref)	-	1.0 (ref)	-	1.0 (ref)	-	1.0 (ref)	-	1.0 (ref)	-
Sex concordance between women	0.74 (0.49, 1.09)	.13	0.59 (0.40, 0.85)	.006	0.87 (0.60, 1.27)	.48	0.76 (0.52, 1.11)	0.16	0.96 0.66, 1.40)	.83	0.90 (0.62, 1.29)	.56	1.02 (0.70, 1.28)	.93
Student URM Status														
Non-URM	1.0 (ref)	-	1.0 (ref)	-	1.0 (ref)	-	1.0 (ref)	-	1.0 (ref)	-	1.0 (ref)	-	1.0 (ref)	-
URM	0.50 (0.25, 0.99)	.006	0.38 (0.19, 0.77)	.007	0.47 (0.26, 0.88)	.02	0.67 (0.37, 1.22)	.19	0.62 (0.37, 1.06)	.08	0.78 (0.60, 1.27)	.34	0.78 (0.47, 1.30)	.35
Interaction between rater and student URM-status														
Non-URM concordance	1.0 (ref)	-	1.0 (ref)	-	1.0 (ref)	-	1.0 (ref)	-	1.0 (ref)	-	1.0 (ref)	-	1.0 (ref)	-
URM concordance	2.92 (0.70, 12.23)	.14	3.97 (0.74, 21.30)	.11	4.38 (0.99, 19.35)	.05	3.47 (0.72, 16.64)	.12	4.56 (1.21, 17.09)	.03	1.28 (0.33, 4.93)	.72	2.60 (0.65, 10.41)	.18

*OR*, odds ratio; *NCAT-EM*, National Clinical Assessment Tool for Emergency Medicine; *URM*, under-represented in medicine.

**Table 5:** Multivariate associations between ratings for National Clinical Assessment Tool for Emergency Medicine domains and Black vs non-Black race, after adjusting for rater sex and race (Black vs non-Black), concordance of student-rater race, clinical site, and time.

	Global assessment		History/physical		Prioritized differential		Ability to formulate plan		Observation/monitoring		Emergency management		Communication	

Variable	OR (95% CI)	P	OR (95% CI)	P	OR (95% CI)	P	OR (95% CI)	P	OR (95% CI)	P	OR (95% CI)	P	OR (95% CI)	P
Student Race														
Non-Black	1.0 (ref)	-	1.0 (ref)	-	1.0 (ref)	-	1.0 (ref)	-	1.0 (ref)	-	1.0 (ref)	-	1.0 (ref)	-
Black	0.67 (0.28, 1.62)	.38	0.32 (0.14, 0.76)	.01	0.36 (0.17, 0.79)	.01	0.46 (0.59, 1.48)	.05	0.54 (0.28, 1.04)	.07	0.78 (0.42, 1.44)	.42	0.65 (0.34, 1.24)	.20
Interaction between Black Raters and Black Students	25.8 (3.23, 205.1)	.002	39.4 (3.42, 452.5)	.003	38.9 (4.56, 331.4)	.001	32.9 (3.40, 317.43)	.003	29.37 (4.55, 189.46)	<.001	2.07 (0.31, 13.9)	.45	17.59 (2.29, 135.41)	.006

*OR*, odds ratio; *NCAT-EM*, National Clinical Assessment Tool for Emergency Medicine.

## References

[1] Cook DA, Beckman TJ (2006). Current concepts in validity and reliability for psychometric instruments: theory and application. Am J Med.

[2] Lawson L, Jung J, Franzen D (2016). Clinical assessment of medical students in emergency medicine clerkships: a survey of current practice. J Emerg Med.

[3] Negaard M, Assimacopoulos E, Harland K (2018). Emergency medicine residency selection criteria: an update and comparison. AEM Educ Train.

[4] Katzung KG, Ankel F, Clark M (2019). What do program directors look for in an applicant?. J Emerg Med.

[5] Jung J, Franzen D, Lawson L (2018). The National Clinical Assessment Tool for Medical Students in the emergency department (NCAT-EM). West J Emerg Med.

[6] Hiller K, Jung J, Lawson L (2021). Multi-institutional implementation of the national clinical assessment tool in emergency medicine: data from the first year of use. AEM Educ Train.

[7] Gauer JL, Mustapha T, Violato C (2024). Race and gender bias in clerkship grading. Teach Learn Med.

[8] Nguemeni Tiako MJ, Ray V, South EC (2022). Medical schools as racialized organizations: how race-neutral structures sustain racial inequality in medical education—a narrative review. J Gen Intern Med.

[9] Winfield A, Amin DP (2022). To the Editor: Where Is equity in the SLOE?. J Grad Med Educ.

[10] Calles I (2023). To the Editor: For equity in assessment: a comment on bias in the emergency medicine Standardized Letter of Evaluation. J Grad Med Educ.

[11] Kukulski P, Ahn J (2021). Validity evidence for the emergency medicine Standardized Letter of Evaluation. J Grad Med Educ.

[12] Colson ER, Pérez M, Blaylock L (2020). Washington University School of Medicine in St. Louis Case Study: A Process for Understanding and Addressing Bias in Clerkship Grading. *Acad* Med.

[13] Cheng D, Promes S, Clem K (2006). Chairperson and faculty gender in academic emergency medicine departments. Acad Emerg Med.

[14] Madsen TE, Linden JA, Rounds K (2017). Current status of gender and racial/ethnic disparities among academic emergency medicine physicians. Acad Emerg Med.

[15] Wiler JL, Wendel SK, Rounds K (2022). Salary disparities based on gender in academic emergency medicine leadership. Acad Emerg Med.

[16] Jena AB, Khullar D, Ho O (2015). Sex differences in academic rank in US medical schools in 2014. JAMA.

[17] Bullock JL, Lockspeiser T, Del Pino-Jones A (2020). They don’t see a lot of people my color: a mixed methods study of racial/ethnic stereotype threat among medical students on core clerkships. Acad Med.

[18] Ackerman-Barger K, Boatright D, Gonzalez-Colaso R (2020). Seeking inclusion excellence: understanding racial microaggressions as experienced by underrepresented medical and nursing students. Acad Med.

[19] Ross DA, Boatright D, Nunez-Smith M (2017). Differences in words used to describe racial and gender groups in Medical Student Performance Evaluations. PLoS ONE.

[20] Axelson RD, Solow CM, Ferguson KJ (2010). Assessing implicit gender bias in Medical Student Performance Evaluations. Eval Health Prof.

[21] Hanson JL, Pérez M, Mason HRC (2022). Racial/ethnic disparities in clerkship grading: perspectives of students and teachers. Acad Med.

[22] O’Sullivan L, Kagabo W, Prasad N (2023). Racial and ethnic bias in medical school clinical grading: a review. J Surg Educ.

[23] Lucey CR, Hauer KE, Boatright D (2020). Medical education’s wicked problem: achieving equity in assessment for medical learners. Acad Med.

[24] Kakara Anderson HL, Govaerts M (2025). Clarifying and expanding equity in assessment by considering three orientations: Fairness, inclusion and justice. Med Educ.

[25] Takeshita J, Wang S, Loren AW (2020). Association of racial/ethnic and gender concordance between patients and physicians with patient experience ratings. JAMA Netw Open.

[26] McOwen KS, Bellini LM, Guerra CE (2007). Evaluation of clinical faculty: gender and minority implications. Acad Med.

[27] Nguemeni Tiako MJ, Wages JE, Perry SP (2023). Black medical students’ sense of belonging and confidence in scholastic abilities at historically Black vs predominantly White medical schools: a prospective study. J Gen Intern Med.

[28] Ghanem N, Goldberg DG, Granger E (2024). A critical qualitative study to understand current Black women medical student perspectives on anti-racist reform in US medical education. Med Educ Online.

[29] Boatright D, Anderson N, Kim JG (2022). Racial and ethnic differences in internal medicine residency assessments. JAMA Netw Open.

[30] Low D, Pollack SW, Liao ZC (2019). Racial/ethnic disparities in clinical grading in medical school. Teach Learn Med.

[31] Downing SM (2002). Threats to the validity of locally developed multiple-choice tests in medical education: construct-irrelevant variance and construct underrepresentation. Adv Health Sci Educ Theory Pract.

[32] Tavakol M, Dennick R (2017). The foundations of measurement and assessment in medical education. Med Teach.

[33] Wheeler M, de Bourmont S, Paul-Emile K (2019). Physician and trainee experiences with patient bias. JAMA Intern Med.

[34] Colson ER, Pérez M, Chibueze S (2023). Understanding and addressing bias in grading: progress at Washington University School of Medicine. Acad Med.

[35] Osseo-Asare A, Balasuriya L, Huot SJ (2018). Minority resident physicians’ views on the role of race/ethnicity in their training experiences in the workplace. JAMA Netw Open.

[36] Heath JK, Dine CJ, LaMarra D (2021). The impact of trainee and standardized patient race and gender on internal medicine resident communication assessment scores. J Grad Med Educ.

[37] Berg K, Blatt B, Lopreiato J (2015). Standardized patient assessment of medical student empathy: ethnicity and gender effects in a multi-institutional study. Acad Med.

[38] Pololi L, Conrad P, Knight S (2009). A Study of the relational aspects of the culture of academic medicine. Acad Med.

[39] Dayal A, O’Connor DM, Qadri U (2017). Comparison of male vs female resident milestone evaluations by faculty during emergency medicine residency training. JAMA Intern Med.

[40] Santen SA, Yamazaki K, Holmboe ES (2020). Comparison of male and female resident milestone assessments during emergency medicine residency training: a national study. Acad Med.

[41] Kogan JR, Dine CJ, Conforti LN (2023). Can rater training improve the quality and accuracy of workplace-based assessment narrative comments and entrustment ratings? A randomized controlled trial. Acad Med.

