# Supplementary material for: Inequities in the National Clinical Assessment Tool for Medical Students in the Emergency Department
Source: West J Emerg Med. 2025 Oct 3;26(5):1250–9. doi: 10.5811/westjem.43506 (PMC12591648; doi:10.5811/westjem.43506)
Supplement: Supplementary file 1 [file wjem-26-1250-s001.docx]

**Supplemental Table 1. NCAT-EM assessment form**

**Did you give in-person feedback?**

| Yes | No |
| --- | --- |

**Your name (evaluator):**

|  |
| --- |

**Shift site:**

| HUP | PPMC | PAH | CHOP | VA | HUP-Cedar |
| --- | --- | --- | --- | --- | --- |

**Focused history and physical exam skills**

| Pre-Entrustable  Extraneous or insufficient information. May miss key physical findings or examine incorrectly. |
| --- |
| Mostly Entrustable  Generally adequate information. Exam mostly adequate and correct. May not differentiate important from extraneous detail. |
| Fully Entrustable/Milestone 1  Appropriate information for clinical content. Exam complete and appropriately tailored. May include excess detail, but thorough and accurate. |
| Outstanding/Milestone 2  Exceptional focused H&P obtains all relevant information. Addresses chief complaint and urgent issues. Differentiates important from extraneous detail. |
| Unable to assess |

**Ability to generate a prioritized differential diagnosis**

| Pre-Entrustable  Limited ability to filter, prioritize, and connect information to generate a basic differentiate based on clinical data and medical knowledge. |
| --- |
| Mostly Entrustable  Generally able to filter and connect information to generate a basic differential based on clinical data and medical knowledge. Beginning to incorporate data and prioritize. |
| Fully Entrustable/Milestone 1  Reliably synthesizes data into a complete differential. Incorporates data. Prioritizes differential by likelihood. |
| Outstanding/Milestone 2  Demonstrates exceptional differential diagnosis and data interpretation. Uses all available information to develop a prioritized differential focusing on life/limb threats. |
| Unable to assess |

**Ability to formulate plan (diagnostic, therapeutic, disposition)**

| Pre-Entrustable  Difficulty applying knowledge to formulate plans or does not offer plan. |
| --- |
| Mostly Entrustable  Usually able to apply knowledge to formulate plans, though plans may be incomplete/incorrect in some details. |
| Fully Entrustable/Milestone 1  Reliably able to apply knowledge to formulate plans that are complete, appropriate, and tailored to patient needs/desires. |
| Outstanding/Milestone 2  Exceptional ability to apply knowledge to formulate outstanding patient-centered plans. |
| Unable to assess |

**Observation, monitoring, and follow-up**

| Pre-Entrustable  May not re-evaluate patients or follow up results in a timely fashion. |
| --- |
| Mostly Entrustable  Usually re-evaluates patients and follows up results, though may need prompting. Beginning to integrate new data into ongoing plan. |
| Fully Entrustable/Milestone 1  Reliably re-evaluates patients and follows up results in a timely manner without prompting. Integrates basic data into ongoing plan, though may need help. Completes tasks despite distraction. |
| Outstanding/Milestone 2  Exceptional re-evaluation and follow up skills. Proactive. Integrates complex results into ongoing plan. Able to handle multiple patients simultaneously. |
| Unable to assess |

**Emergency recognition and management**

| Pre-Entrustable  May not recognize or respond to abnormal vital signs or patient deterioration. Delays or fails to seek help. Unable to recommend stabilization interventions. |
| --- |
| Mostly Entrustable  Recognizes and responds to most abnormal vital signs but may miss subtle changes. Promptly seeks help. Recommends and/or initiates some basic stabilization interventions. |
| Fully Entrustable/Milestone 1  Reliably recognizes and responds to all vital sign abnormalities and trends. Promptly seeks help. Recommends and/or initiates all basic and some advanced stabilization interventions. |
| Outstanding/Milestone 2  Exceptionally attentive to vital sign abnormalities and patient deterioration. Promptly seeks help. Recommends and/or initiates basic and advanced interventions appropriately. |
| Unable to assess |

**Patient- and team-centered communication**

| Pre-Entrustable  Communication with patients and/or team is unidirectional or not tailored to circumstances. May not read or respond to others’ emotions well. May not always attend to patient comfort or preferences. May not always integrate well into team, may not recognize value of team contributions. |
| --- |
| Mostly Entrustable  Communication with patients and/or team is bidirectional and usually tailored to circumstances. Generally, reads and responds to others’ emotions well. Usually attentive to patient comfort and preferences. Usually integrates well into team, may not fully understand team roles or contributions. |
| Fully Entrustable/Milestone 1  Communication with patients and/or team is bidirectional and reliably tailored to circumstances. Skillful in reading and responding to others’ emotions. Reliably sensitive to patient perspective and preferences. Integrates well into team and recognizes value of team members. |
| Outstanding/Milestone 2  Demonstrates exceptional communication skills with patients and/or team. Effectively reads and negotiates complex emotional situations and conflicts. Always sensitive to patient perspective. Highly regarded by patients and team. |
| Unable to assess |

**Do you have any concerns regarding this student’s professionalism?**

| Yes | No |
| --- | --- |

**If yes, please describe specific behaviors observed:**

|  |
| --- |

**Global assessment - compared to other students with similar level of experience, this student's performance today was:**

| Lower 1/3 |
| --- |
| Middle 1/3 |
| Top 1/3 |
| Exceptional (top 10%) |

**Please comment on 1-2 areas:**

**Strengths:**

|  |
| --- |

**Areas to improve:**

|  |
| --- |
