# Supplementary material for: Inequities in the National Clinical Assessment Tool for Medical Students in the Emergency Department
Source: West J Emerg Med. 2025 Oct 3;26(5):1250–9. doi: 10.5811/westjem.43506 (PMC12591648; doi:10.5811/westjem.43506)
Supplement: Supplementary file 2 [file wjem-26-1250-s002.docx]

**Supplemental Table 2. Multivariable associations between ratings for NCAT-EM domains and student gender and URM-status, after adjusting for rater gender and URM-status, concordance of student-rater URM-status, clinical site, and time**. OR = Odds Ratio. CI = confidence interval. NCAT-EM = National Clinical Assessment Tool for Emergency Medicine. URM = under-represented in medicine.

|  | **Global Assessment** | | **History/Physical** | | **Prioritized Differential** | | **Ability to Formulate Plan** | | **Observation/Monitoring** | | **Emergency Management** | | | **Communication** | | |
| --- | --- | --- | --- | --- | --- | --- | --- | --- | --- | --- | --- | --- | --- | --- | --- | --- |
| Variable | **OR (95% CI)** | **P** | **OR (95% CI)** | **P** | **OR (95% CI)** | **P** | **OR (95% CI)** | **P** | **OR (95% CI)** | **P** | **OR (95% CI)** | **P** | **OR (95% CI)** | | **P** |  |
| **Student Gender** |  |  |  |  |  |  |  |  |  |  |  |  |  | |  |  |
| Men | 1.0 (ref) | - | 1.0 (ref) | - | 1.0 (ref) | - | 1.0 (ref) | - | 1.0 (ref) | - | 1.0 (ref) | - | 1.0 (ref) | | - |  |
| Women | 0.93 (0.54, 1.60) | 0.78 | 0.84 (0.50, 1.42) | 0.52 | 0.98 (0.61, 1.57) | 0.93 | 0.95 (0.60, 1.51) | 0.84 | 1.26 (0.84, 1.89) | 0.26 | 0.87 (0.60, 1.27) | 0.48 | 1.21 (0.82, 1.78) | | 0.35 |  |
| **Student URM-Status** |  |  |  |  |  |  |  |  |  |  |  |  |  | |  |  |
| Non-URM | 1.0 (ref) | - | 1.0 (ref) | - | 1.0 (ref) | - | 1.0 (ref) | - | 1.0 (ref) | - | 1.0 (ref) | - | 1.0 (ref) | | - |  |
| URM | 0.50 (0.25, 0.99) | 0.006 | 0.38 (0.19, 0.77) | 0.007 | 0.47 (0.26, 0.88) | 0.02 | 0.67 (0.37, 1.22) | 0.19 | 0.62 (0.37, 1.06) | 0.08 | 0.78 (0.60, 1.27) | 0.34 | 0.78 (0.47, 1.30) | | 0.35 |  |
| **Interaction Between Rater and Student URM-Status** |  |  |  |  |  |  |  |  |  |  |  |  |  | |  |  |
| Non-URM Concordance | 1.0 (ref) | - | 1.0 (ref) | - | 1.0 (ref) | - | 1.0 (ref) | - | 1.0 (ref) | - | 1.0 (ref) | - | 1.0 (ref) | | - |  |
| URM Concordance | 2.92 (0.70, 12.23) | 0.14 | 3.97 (0.74, 21.30) | 0.11 | 4.38 (0.99, 19.35) | 0.05 | 3.47 (0.72, 16.64) | 0.12 | 4.56 (1.21, 17.09) | 0.03 | 1.28 (0.33, 4.93) | 0.72 | 2.60 (0.65, 10.41) | | 0.18 |  |
